# Energetic Contributions Including Gender Differences and Metabolic Flexibility in the General Population and Athletes

**DOI:** 10.3390/metabo12100965

**Published:** 2022-10-12

**Authors:** Woo-Hwi Yang, Jeong-Hyun Park, So-Young Park, Yongdoo Park

**Affiliations:** 1 Graduate School of Sports Medicine, CHA University, Seongnam-si 13503, Korea; 2 Department of Medicine, General Graduate School, CHA University, Seongnam-si 13503, Korea; 3 Department of Biomedical Engineering, College of Medicine, Korea University, Seoul 02841, Korea

**Keywords:** aerobic performance, fat oxidation, health, lactate, mitochondrial function, oxidative capacity

## Abstract

Metabolic flexibility includes the ability to perform fat and carbohydrate oxidation, as well as oxidative capacity, which is associated with mitochondrial function, energetic contributions, and physical health and performance. During a session of graded incremental exercise testing (GIET), we investigated metabolic flexibility, the contributions of three energy systems, and performances of individuals with different metabolic characteristics. Fifteen general population (GP; *n* = 15, male *n* = 7, female *n* = 8) and 15 national-level half-marathon and triathlon athletes (A; *n* = 15, male *n* = 7, female *n* = 8) participated in this study. During GIET, heart rate (HR), oxygen uptake ($\dot{V}$O_2mean_ and $\dot{V}$CO_2mean_), metabolic equivalents (METs) in $\dot{V}$O_2mean_, and blood glucose and lactate concentrations (La^−^) were measured. Furthermore, jogging/running speeds (S) at specific La^−^, fat and carbohydrate oxidations (FATox and CHOox), and energetic contributions (oxidative; W_Oxi_, glycolytic; W_Gly_, and phosphagen; W_PCr_) were calculated. The percentages of HR_max_, relative $\dot{V}$O_2mean_, $\dot{V}$CO_2mean_, and METs in $\dot{V}$O_2mean_ were all lower in A than they were in GP. FATox values were lower in GP than in A, while CHOox and La^−^ were higher in GP than in A. Negative correlations between La^−^ and FATox were also observed in both groups. Contributions of W_Oxi_, W_Gly_, and W_PCr_ were higher in GP than in A during GIET. Moreover, values of W_Gly_, and W_PCr_ were significantly lower and higher, respectively, in male GP than in female GP. Furthermore, S at specific La^−^ were higher in A than in GP. It is suggested that an individualized low-intensity recovery exercise program be established, to achieve increased metabolic flexibility and oxidative capacity (aerobic base), such as public health improvements and a greater volume of higher exercise intensities; this is the type of exercise that elite athletes worldwide mostly perform during their training period and progression. This may prevent cardiac/metabolic diseases in GP.

## 1. Introduction

Physical inactivity has been shown to cause non-communicable diseases such as metabolic diseases [1,2,3]. Insulin resistance, type 2 diabetes mellitus, obesity, and metabolic syndrome are associated with mitochondrial dysfunction [4,5]. Decreased mitochondrial respiratory capacity as a dysfunction eventually leads to metabolic inflexibility [5,6,7]. Individuals with these diseases exhibit decreases in the subsarcolemmal and interfibrillar areas of the mitochondrial reticulum, such as degraded muscle mitochondrial electron transport chain capacity [5,8]. Lipid and carbohydrate metabolism are related to mitochondrial density and function, which are also crucial factors affecting the capacity for fatty acid metabolism [5,9].

In an incremental cycling test, San-Millán et al. [5] reported that professional endurance athletes utilized higher fat oxidation (0.50–0.67 g∙min^−1^) between 136.5 and 238.8 watts than moderately active individuals and individuals with metabolic syndrome. Furthermore, the blood lactate concentrations (La^−^) of professional endurance athletes remained <1 mmol∙L^−1^ during the given exercise intensities, while the La^−^ of the other groups were between 5.97 and 6.38 mmol∙L^−1^ during the same exercise intensities. The reduced levels of La^−^ in athletes imply increased La^−^ elimination occurring alongside adenosine triphosphate (ATP) re-synthesis, lipid oxidation, gluconeogenesis, and decreases in both glucose and total carbohydrate use [5,10,11].

Lactate is the main energy source, a main gluconeogenic precursor, a regulator of intermediary metabolism, and a signal molecule [5,10,12,13]. During lactate shuttle for production and elimination, lactate induces fat and carbohydrate metabolism [4,5,10]. Fat and carbohydrate oxidations, including glucose and lactate, also play key roles in cardiac metabolism [13]. These metabolic responses, such as oxidative exercise capacity, are associated with the cardiovascular system/health [14]. The produced lactate is re-metabolized via mitochondrial lactate oxidation, which involves monocarboxylic transporter-1, its chaperone as the cluster of differentiation 147 (CD147), which is an ancillary protein, as well as mitochondrial dehydrogenase and cytochrome oxidase [5,10,13,15]. In previous studies, measured lactate and carbohydrate and fat oxidation have been indirect indicators of mitochondrial function and metabolic flexibility [5,10,16]. Furthermore, lactate is a simple, efficient, and fast indirect parameter for the calculation of substrate use as mitochondrial function [5,10,16]. Previous studies have only indicated La^−^ and oxygen uptake ($\dot{V}$O_2_) levels, and fat and carbohydrate oxidation using stoichiometry in different individuals, which are related to indirect indicators of metabolic flexibility during incremental cycling tests [5,16,17]. Furthermore, an analysis of energetic contributions provides information about the physiological responses that are calculated using assessed $\dot{V}$O_2_ and La^−^ values during different exercises in mathematical models [18,19,20,21]. Metabolic energy contributions during exercise are crucial aspects of physiological performance, and these are required for a better understanding of metabolic reactions, to enhance exercise periodization and methods for different individuals [21,22]. To date, there has been a lack of direct analyses and comparisons of the three energy systems in terms of gender differences and differences between the general population and athletes, and there is a need for research examining the three energy system contributions (oxidative, glycolytic, and phosphagen) during jogging and running. 

Therefore, this study aimed to investigate energetic contributions including gender differences, metabolic flexibility, and performance parameters, and to compare how these abilities differ between the general population and athletes, during graded incremental jogging/running tests. These evaluations are expected to support an efficient method of individualized training prescriptions to improve physical performances in order to prevent cardiac/metabolic diseases in the general population, as they have proven useful worldwide in enhancing physical health and performance in elite athletes [5].

## 2. Materials and Methods

### 2.1. Participants

The sample size was calculated and considered, based on previous studies [2,5,16,18,20,21,23,24]: effect size: 1.10, alpha error probability: 0.05, and statistical power: 0.80 (G*Power software, version 3.1.9.4; Heinlich Heine University, Düsseldorf, Germany). The total required sample size was estimated to be thirty participants (*n* = 30). Fifteen physically active male and female individuals from the general population (*n* = 15; male: 7 and female: 8, general population group, GP) and 15 national-level male and female half-marathon and triathlon athletes (*n* = 15; male: 7 and female: 8, athletes’ group, A) participated in this study. Table 1 presents the anthropometric data of all participants. The participants in the GP were involved in running and resistance training for at least 4 to 6 h per week, while the athletes trained for at least 16 to 20 h per week. Participants completed an anthropometric test using an 8-electrode segmental multifrequency bioelectrical impedance analysis (InBody 270; InBody Co., Ltd., Seoul, Korea). Participants were instructed not to take any medication on the test day and to abstain from alcohol and nicotine consumption for at least 24 h before the test. The study was approved by the Institutional Review Board of CHA University (No. 1044308-202010-HR-045-02). The applied guidelines align with the Declaration of Helsinki. All participants provided written informed consent.

### 2.2. Graded Incremental Exercise Testing

The graded incremental exercise test (GIET) was conducted on a treadmill (NR30XA, DRAX Corporation Ltd., Seoul, Republic of Korea) with 5-min steps interspersed with 30 s breaks between steps for La^−^ measurements. The initial jogging speed was 1.5 m∙s^−1^, which was increased to 0.5 m∙s^−1^ every five minutes. The GIET was stopped when La^−^ exceeded 4 mmol∙L^−1^ after each jogging/running speed in all participants [16,17,25]. Capillary blood sampling for lactate and glucose determination was taken from the earlobe (20 µL) immediately after each 5-min step. The La^−^ and glucose levels for all steps were analyzed using an enzymatic–amperometric sensor chip system (Biosen C-line, EKF diagnostics sales GmbH, Barleben, Germany). Heart rate (HR) data were recorded using a Polar H10 sensor (Polar Electro, Kemple, Finland). The average HR value over the last 30 s of each step was estimated for statistical analyses [16]. The percentages of estimated maximal HR (HR_max_) were calculated using previously described methods [16,26]. The jogging/running speed (S) and HR at 1.5, 2.0, 3.0, and 4.0 mmol∙L^−1^ La^−^ were analyzed using a previously suggested mathematical model of interpolation [16,17,27]. During GIET, oxygen uptake ($\dot{V}$O_2_, $\dot{V}$O_2mean_, metabolic equivalents; METs in $\dot{V}$O_2mean_ [2,28], and carbon dioxide; $\dot{V}$CO_2mean_) was measured breath-by-breath, using a mobile gas analyzer MetaMax 3B (Cortex Biophysik, Leipzig, Germany). The gas analyzer was calibrated with calibration gas (15% O_2_ and 5% CO_2_, Cortex Biophysik, Leipzig, Germany), and the turbine volume transducer was calibrated with a 3-L syringe (Hans Rudolph, Kansas City, MO, USA).

### 2.3. Calculations of Fat and Carbohydrate Oxidation Rate during GIET

During GIET, $\dot{V}$O_2_ and $\dot{V}$CO_2_ production were used to calculate metabolic flexibility as well as fat (FATox) and carbohydrate (CHOox) oxidation, using stoichiometric equations as described in previous studies [5,24,29]:

$$\mathrm{FATox}\left(g\cdot \min^{-1}\right):1.67\cdot {\dot{V}O}_{2}\left(L\cdot \min^{-1}\right)-1.67\cdot {\dot{V}\mathrm{CO}}_{2}\left(L\cdot \min^{-1}\right)$$

$$\mathrm{CHOox}\left(g\cdot \min^{-1}\right):4.55\cdot {\dot{V}\mathrm{CO}}_{2}\left(L\cdot \min^{-1}\right)-3.21\cdot {\dot{V}O}_{2}\left(L\cdot \min^{-1}\right)$$

### 2.4. Calculations of Energetic Contributions during GIET

Energetic contributions in kilojoules (kJ), such as contributions in the oxidative (W_Oxi_), glycolytic (W_Gly_), and phosphagen (W_PCr_) systems, were estimated by measurements of $\dot{V}$O_2_ during GIET, La^−^ after each step of GIET, and the fast phase of excess $\dot{V}$O_2_ after exercise [2,18,20,21].

The W_Oxi_ was calculated by subtracting $\dot{V}$O_2_ in the rest steps from $\dot{V}$O_2_ during the exercise steps by the trapezoidal method, where the area under the curve was divided into sections, and then the sum of the trapezoid was used to calculate the integral [20,21]. The value of $\dot{V}$O_2rest_ was determined in the standing position on a treadmill, with the last 30 s of a 5 min phase used as a reference [2,18,20,21].

The W_Gly_ was estimated as La^−^ levels after each step of GIET, assuming that the production of 1 mmol∙L^−1^ is equivalent to 3 mL O_2_∙kg^−1^ of body mass [19]. The difference (Δ) in La^−^ was calculated by subtracting La^−^ at the previous step from La^−^ after the exercise step (only ΔLa^−^ at 1.5 m∙s^−1^, resting La^−^ was subtracted) [18,20,21].

The W_PCr_ was calculated by considering $\dot{V}$O_2_ during the interval between graded incremental steps and the fast component of excess post-exercise after the last step of GIET [18,20,21]. The *off* $\dot{V}$O_2_ kinetics were fitted by mono-exponential and bi-exponential models using OriginPro 2021 (OriginLab Corp., Northampton, UK). The slow component of the bi-exponential model was negligible. Therefore, the post-exercise $\dot{V}$O_2_ values were fitted to a mono-exponential model, and W_PCr_ was obtained by calculating the amplitude and time constant of the exponential area [2,18,19,20,21]. The caloric quotient of 20.92 kJ was used in all three absolute energetic contributions [23]. The total energy expenditure was estimated as the sum of the three energy systems (W_Oxi_, W_Gly_, and W_PCr_) in kJ. The contribution of the three energy systems was indicated as a percentage (%) related to total energy expenditure.

### 2.5. Statistical Analyses

All parameters were analyzed using GraphPad Prism 9.4.0. (GraphPad Prism Software Inc., La Jolla, CA, USA). Data are presented as mean ± standard deviation (SD) and standard error of the mean (S.E.M.). The normal distribution (ND) of all data was conducted using the Shapiro−Wilk test. During GIET, both groups were compared statistically up to 3.5 m∙s^−1^ steps, because the GP group was able to perform their test until the 3.5 m∙s^−1^ steps. After the ND test, HR at 3.0 m∙s^−1^; La^−^ at 1.5 and 3.0 m∙s^−1^; % of HR_max_ at 3.0 m∙s^−1^; relative $\dot{V}$O_2mean_ at 2.0 m∙s^−1^; METs in $\dot{V}$O_2mean_ at 1.5 and 2.0 m∙s^−1^; relative $\dot{V}$CO_2mean_ at 1.5, 2.0, and 3.0 m∙s^−1^; FATox at 3.0 and 3.5 m∙s^−1^; W_PCr_ in kJ at 1.5 m∙s^−1^; W_Gly_ in kJ at 1.5 and 3.0 m∙s^−1^; W_PCr_ in % at 2.0 m∙s^−1^; W_Gly_ in % at 1.5, 2.0, 2.5, and 3.0 m∙s^−1^; and W_Oxi_ in % at 2.0 m∙s^−1^ steps were compared, using a Mann-Whitney-U rank test (non-parametric test). The remaining data were analyzed using an unpaired *t*-test (parametric test). In addition, the average values up to 3.5 m∙s^−1^ steps during GIET of the three energy systems and the total energy demand between males (*n* = 7) and females (*n* = 8) in GP and A were statistically compared, using the Mann-Whitney-U rank test. The alpha level of significance was set at *p* < 0.05 for all tests. The effect size (ES, Cohen’s *d* and Z√N; *d* and *r*) was estimated for parametric and non-parametric tests. The thresholds for small, medium, and large effects were 0.2, 0.5, and 0.8, respectively (parametric test), and 0.1, 0.3, and 0.5, respectively (non-parametric test) [30]. Furthermore, Pearson’s two-tailed correlation and linear regression analyses were performed between La^−^ and FATox in GP and A.

## 3. Results

### 3.1. Comparisons of Physiological Parameters, Metabolic Flexibility (FATox and CHOox), and Correlation and Regression Analyses between La^−^ and FATox

During all steps of GIET, the absolute values of $\dot{V}$O_2mean_, $\dot{V}$CO_2_, and blood glucose between both groups were not significantly different (*p* > 0.05). Only the value of HR at 3.0 m∙s^−1^ was significantly higher in GP than in A (*p* = 0.0319; ES [*r*]: −0.4) (Table 2). Furthermore, the percentages of HR_max_ at 2.5 and 3.0 m∙s^−1^ were significantly lower in A than in GP (*p* = 0.0299; ES [*d*]: 0.9, *p* = 0.0167; ES [*r*]: −0.4, respectively). The relative values of $\dot{V}$O_2mean_ from 1.5 to 3.0 m∙s^−1^ were also significantly lower in A than in GP (*p* = 0.0079; ES [*d*]: 1.0, *p* = 0.0007; ES [*r*]: −0.6, *p* = 0.0036; ES [*d*]: 1.2, *p* = 0.0068; ES [*d*]: 1.1, respectively). Moreover, the values of METs in $\dot{V}$O_2mean_ from 1.5 to 3.0 m∙s^−1^ were significantly lower in A than in GP (*p* = 0.0063; ES [*r*]: −0.5, *p* = 0.0009; ES [*r*]: −0.6, *p* = 0.0037; ES [*d*]: 1.2, *p* = 0.0068; ES [*d*]: 1.1, respectively). Lastly, the relative levels of $\dot{V}$CO_2mean_ from 1.5 to 3.5 m∙s^−1^ were significantly lower in A than in GP (*p* = 0.0367; ES [*r*]: −0.4, *p* = 0.0016; ES [*r*]: −0.6, *p* = 0.0008; ES [*r*]: −0.6, *p* = 0.0020; ES [*d*]: 1.2, *p* = 0.0378; ES [*d*]: 0.9, respectively) (Table 2).

**Figure 1:**
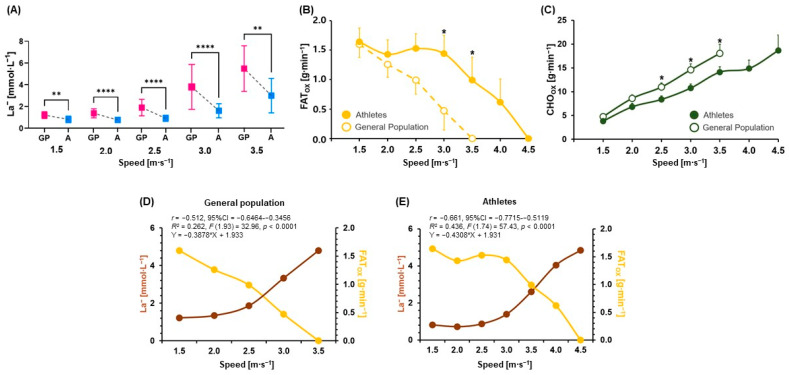
(**A**) La^−^ at 1.5, 2.0, 2.5, 3.0, and 3.5 speeds of GIET between GP and A, (**B**) FATox between GP and A during GIET, (**C**) CHOox between GP and A during GIET, (**D**) correlation and regression analyses between La^−^ and FATox in GP, and (**E**) correlation and regression analyses between La^−^ and FATox in A. Significant differences (* *p* < 0.05, ** *p* < 0.01, **** *p* < 0.0001). A: athletes; CHOox: carbohydrate oxidation; FATox: fat oxidation; GP: the general population; GIET: graded incremental exercise test; La^−^: blood lactate concentrations. Data are mean ± standard error of the mean (S.E.M.) (**A**–**C**).

La^−^ at 1.5, 2.0, 2.5, 3.0, and 3.5 m∙s^−1^ were significantly higher in GP than in A (*p* = 0.0016; ES [*r*]: −0.6, *p* < 0.0001; ES [*d*]: 1.8, *p* < 0.0001; ES [*d*]: 1.7, *p* < 0.0001; ES [*r*]: −0.7, *p* = 0.0017; ES [*d*]: 1.3, respectively) (Table 2 and Figure 1A). Moreover, the levels of FATox at 3.0 and 3.5 m∙s^−1^ were significantly lower in GP than in A (*p* = 0.0141; ES [*r*]: −0.6, *p* = 0.0159; ES [*r*]: −0.6, respectively), while the values of CHOox at 2.5, 3.0, and 3.5 m∙s^−1^ were significantly higher in GP than in A (*p* = 0.0304; ES [*d*]: 0.8, *p* = 0.0155; ES [*d*]: 0.9, *p* = 0.0237; ES [*d*]: 0.9, respectively) (Table 2 and Figure 1B,C).

Moderate negative correlations and linear regressions were observed between La^−^ and FATox in GP and A (*r* = −0.512; 95% confidence interval [CI]: −0.6464–−0.3456; *R*^2^ = 0.262; *F* (1.93) = 32.96; *p* < 0.0001, *r* = −0.661; 95%CI: −0.7715–−0.5119; *R*^2^ = 0.436; *F* (1.74) = 57.43; *p* < 0.0001, respectively) (Figure 1D,E).

### 3.2. Jogging/Running Speeds and HR at Certain La^−^

**Figure 2:**
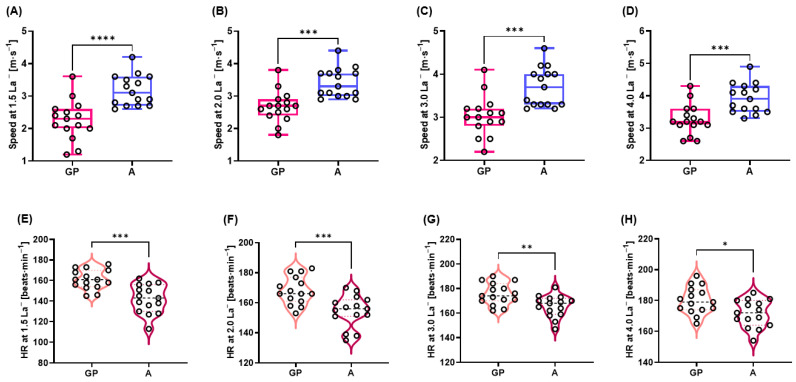
Jogging/running S at (**A**) 1.5 mmol∙L^−1^ La^−^, (**B**) 2.0 mmol∙L^−1^ La^−^, (**C**) 3.0 mmol∙L^−1^ La^−^, (**D**) 4.0 mmol∙L^−1^ La^−^, HR at (**E**) 1.5 mmol∙L^−1^ La^−^, (**F**) 2.0 mmol∙L^−1^ La^−^, (**G**) 3.0 mmol∙L^−1^ La^−^, and (**H**) 4.0 mmol∙L^−1^ La^−^. * *p* < 0.05, ** *p* < 0.01, *** *p* < 0.001 **** *p* < 0.0001. A: athletes; HR: heart rate; GP: general population; S: speeds.

The jogging/running speeds from 1.5 to 4.0 mmol∙L^−1^ La^−^ were significantly higher in A than GP, while the HR values from 1.5 to 4.0 mmol∙L^−1^ La^−^ were significantly higher in GP than A (speeds: *p* < 0.0001; ES [*d*]: 1.6, *p* = 0.0002; ES [*d*]: 1.5, *p* = 0.0004; ES [*d*]: 1.4, *p* = 0.0006; ES [*d*]: 1.4, HR: *p* = 0.0002; ES [*d*]: 1.6, *p* = 0.0007; ES [*d*]: 1.4, *p* = 0.0047; ES [*d*]: 1.2, *p* = 0.0130; ES [*d*]: 1.1, respectively) (Figure 2A–H).

### 3.3. Mean energetic contributions until 3.5 m∙s^−1^ Steps during GIET between Males and Females in GP and A

**Figure 3:**
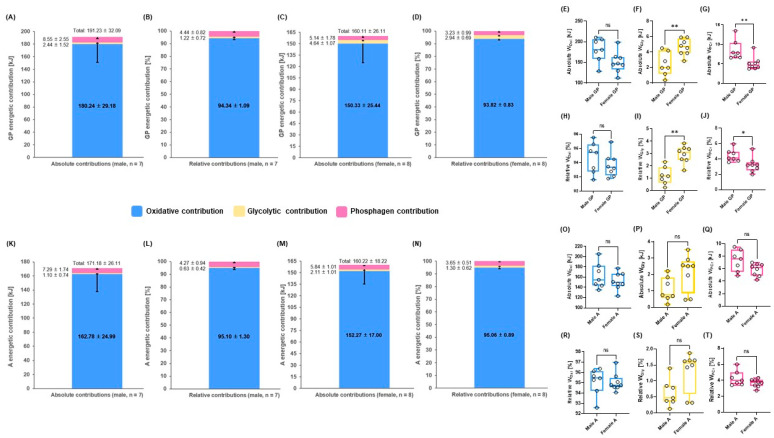
Comparisons of mean energetic contributions until 3.5 m∙s^−1^ steps during GIET between males and females in GP and A. (**A**) Overview of the three energy systems in male GP (kJ), (**B**) in male GP (%), (**C**) female GP (kJ), (**D**) female GP (%), (**E**) comparison of absolute oxidative contribution (kJ) between male and female GP, (**F**) glycolytic contribution (kJ) between male and female GP, (**G**) phosphagen contribution (kJ) between male and female GP; (**H**) comparison of relative oxidative contribution (%) between male and female GP, (**I**) glycolytic contribution (%) between male and female GP, (**J**) phosphagen contribution (%) between male and female GP; (**K**) overview of the three energy systems in male A (kJ), (**L**) in male A (%), (**M**) in female A (kJ), (**N**) in female A (%); (**O**) comparison of absolute oxidative contribution (kJ) between male and female A, (**P**) glycolytic contribution (kJ) between male and female A, (**Q**) phosphagen contribution (kJ) between male and female A; (**R**) comparison of relative oxidative contribution (%) between male and female A, (**S**) glycolytic contribution (%) between male and female A, and (**T**) phosphagen contribution (%) between male and female A. ns: *p* > 0.05, * *p* < 0.05, ** *p* < 0.01. A: athletes; GP: the general population; W_Oxi_: oxidative system contribution; W_Gly_: glycolytic system contribution; W_PCr_: phosphagen system contribution. Male GP (*n* = 7), female GP (*n* = 8), male A (*n* =7), and female A (*n* = 8). Data are mean ± standard deviation (SD) (A–D and K–N).

The mean values of energetic contributions during GIET until 3.5 m∙s^−1^ showed that absolute (kJ) W_Gly_ in male GP was significantly lower than that in female GP (*p* = 0.0093; ES [*r*]: −0.6), while W_PCr_ (kJ) in male GP was significantly higher than that in female GP (*p* = 0.0059; ES [*r*]: −0.7). Moreover, similar results were found in the relative (%) energetic contributions (*p* = 0.0012; ES [*r*]: −0.8, *p* = 0.0140; ES [*r*]: −0.6) There were no significant differences in energetic contributions between males and females in A and total energy demand between males and females in GP and A (*p* > 0.05) (Figure 3A–T).

### 3.4. Energetic Contributions between GP and A during GIET

**Figure 4:**
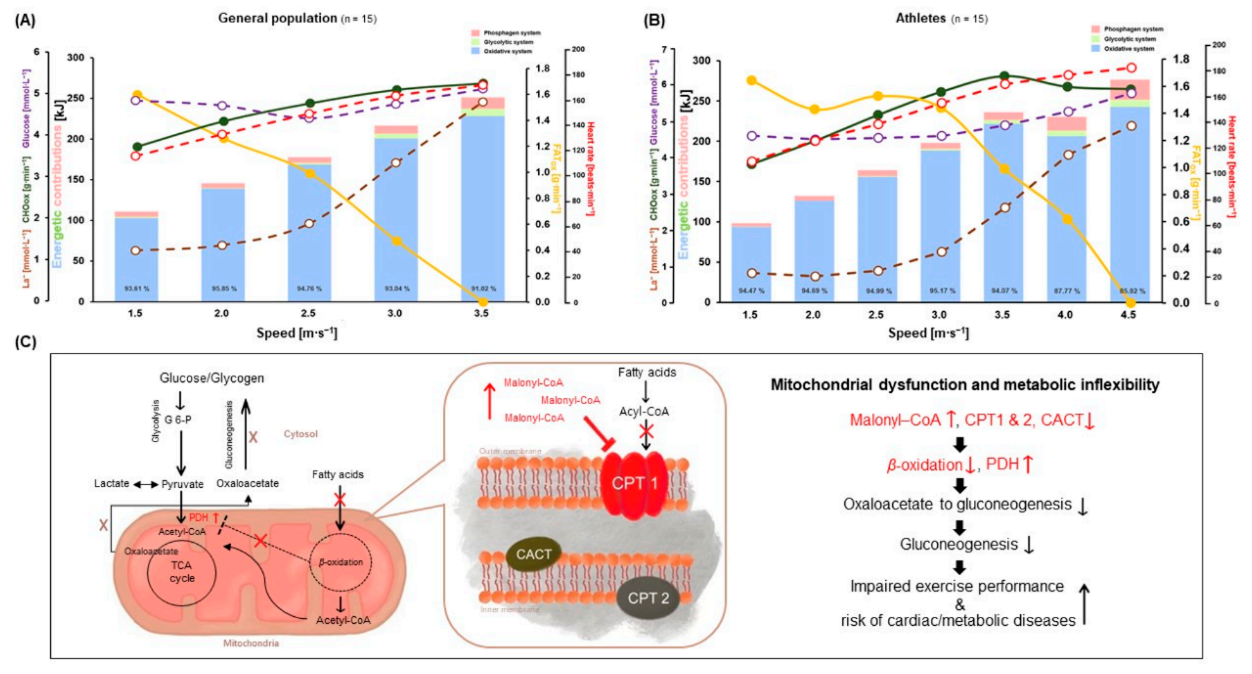
(**A**) FATox and CHOox oxidations, energetic contributions, blood glucose, La^−^, HR in GP during GIET, (**B**) FATox and CHOox oxidations, energetic contributions, blood glucose, La^−^, HR in A during GIET, and (**C**) Schematic representation of inhibited fat metabolism such as metabolic inflexibility. A: athletes; CACT: carnitine-acylcarnitine-translocase; CPT: carnitine palmitoyltransferase; CHOox: carbohydrate oxidation; CoA: Coenzyme A; FATox: fat oxidation; GP: general population; GIET: graded incremental exercise test; G 6-P: glucose 6-phosphate; La^−^: blood lactate concentrations; PDH: pyruvate dehydrogenase; TCA: tricarboxylic acid.

Regarding energetic contributions, only W_PCr_ in kJ at 1.5 m∙s^−1^ was significantly higher in GP than A (*p* = 0.0186; ES [*r*]: −0.1), while W_Gly_ in kJ from 1.5 to 3.5 m∙s^−1^ was significantly higher in GP than A (*p* = 0.0002; ES [*r*]: −0.6, *p* = 0.0107; ES [*d*]: 0.9, *p* = 0.0046; ES [*d*]: 1.1, *p* = 0.0006; ES [*r*]: −0.6, *p* = 0.0165; ES [*d*]: 0.9, respectively). W_Oxi_ in kJ showed no significant difference between both groups. Furthermore, the relative energetic contributions (%) showed that only W_PCr_ at 2.0 m∙s^−1^ was significantly lower in GP than A (*p* = 0.0030; ES [*r*]: −0.5). Moreover, W_Gly_ in % from 1.5 to 3.5 m∙s^−1^ showed significantly higher values in GP than A (*p* = 0.0003; ES [*r*]: −0.6, *p* = 0.0157; ES [*r*]: −0.5, *p* = 0.0068; ES [*r*]: −0.5, *p* = 0.0048; ES [*r*]: −0.5, *p* = 0.0229; ES [*d*]: 0.9, respectively). W_Oxi_ at 2.0 m∙s^−1^ was significantly higher in GP than A (*p* = 0.0270; ES [*r*]: −0.4) while W_Oxi_ at 3.0 m∙s^−1^ was significantly lower in GP than A (*p* = 0.0069; ES [*d*]: 1.0) (Table 2 and Figure 4A,B).

## 4. Discussion

Physiological variables, metabolic flexibility, and three energy systems during the graded incremental jogging/running test were compared between GP and A. Moreover, the mean energetic contributions up to 3.5 m∙s^−1^ steps during GIET were compared between males and females in GP and A. To our knowledge, this study is the first to compare the FATox and CHOox, combining three energy system contributions while including gender differences during jogging/running GIET between both groups. Compared with GP, lower percentages of HR_max_, relative $\dot{V}$O_2mean_ and $\dot{V}$CO_2mean_, and METs in $\dot{V}$O_2mean_ were observed in A. The values of La^−^, CHOox, W_Oxi_, W_Gly_, and W_PCr_ were found to be higher in GP than in A, including lower and higher mean absolute and relative values of W_Gly_ and W_PCr_ were found in male GP than in female GP, respectively, while FATox was higher in A than in GP. Both groups showed predominant usage of the oxidative energy system and moderate negative correlations between La^−^ and FATox.

There was a significant difference in HR at 3.0 m∙s^−1^ only. However, the values of HR in A during GIET tended to be lower than in GP, and the HR values at certain La^−^ in A were lower than in GP. Furthermore, the percentages of HR_max_, relative $\dot{V}$O_2mean_ and $\dot{V}$CO_2mean_, and METs in $\dot{V}$O_2mean_ were all lower in A than in GP, thus indicating that athletes performed at a relatively lower exercise intensity compared to GP during the same absolute exercise intensity. These phenomena might be associated with so-called “athlete’s heart” [31]. Cardiovascular responses to exercise vary, according to activity type [31]. For instance, middle- and long-distance running increases HR, cardiac output, and stroke volume, decreases peripheral vascular resistance, and modestly increases blood pressure, all of which affect the volume load on the left ventricle [31,32,33]. Therefore, previous studies have reported that (maximal) left ventricular wall thickness and end-diastolic diameter, such as left ventricular hypertrophy were greater in athletes than non-athletes [31,34,35,36]. However, the results of this study should be further explored with the additional HR- and $\dot{V}$O_2_-related parameters mentioned above.

The La^−^ levels were lower in A, in which, in turn, FATox was higher than it was in GP during GIET. This is attributable to the greater lactate elimination in A than in GP [5,10]. Measuring La^−^ and FATox during exercise represents an alternative approach to reflecting mitochondrial function and metabolic flexibility in ambulatory and clinical settings [5]. Recent studies suggest that GP and A with relatively poor endurance capabilities cannot efficiently oxidize fat during low to moderate exercise [5,10,16]. Accordingly, they use more carbohydrates and produce higher La^−^, while the uptake of free fatty acids can be inhibited by accumulated malonyl-Coenzyme A (CoA), which decreases carnitine palmitoyltransferase 1 (CPT1) activation, beta (*ß*)-oxidation in mitochondria, and gluconeogenesis [5,10,37]. Beyond the difficulties of fatty acid oxidation in the form of mitochondrial dysfunction in individuals with metabolic syndrome, an impaired ability to eliminate pyruvate and lactate by oxidation and gluconeogenesis affect the formation of malonyl-CoA, which is an inhibitor of CPT1 and decreases mitochondrial fatty acid uptake and oxidation (Figure 4C) [5,10,38]. During lactate re-metabolism, such as at low-exercise intensities, fatty acid derivatives are oxidized in mitochondria and can increase acetyl-CoA [5,10,39]. Furthermore, *ß*-oxidation in mitochondria inhibits pyruvate dehydrogenase (PDH). However, the tricarboxylic acid cycle is substituted, to enable continued anaplerosis and pyruvate carboxylase (PC), because inhibited PDH is the major anaplerotic enzyme that immediately synthesizes oxaloacetate from pyruvate [10,39]. This anaplerotic mechanism is necessary for gluconeogenesis, lipogenesis, and ATP re-synthesis from lactate during low-intensity exercise in well-trained athletes [5,10,21,40]. FATox activates crucial enzymes and hormonal responses of gluconeogenesis such as pyruvate kinase, PC, phosphoenolpyruvate carboxykinase, epinephrine, glucagon, cortisol, and other related regulators such as cyclic adenosine monophosphate and intracellular Ca^+^ handling [10,16,17]. In these regards, comparisons of CHOox between both groups and negative correlation and regression analyses between La^−^ and FATox support these mechanisms and the differences in mitochondrial function between both groups [5]. Higher CHOox in GP showed increased contributions of absolute and relative W_Gly_ compared with A during GIET. Moreover, different values of W_Oxi_ in % were affected by higher W_PCr_ and W_Gly_ in kJ. However, there were no differences in absolute W_Oxi_ and W_TOTAL_ between both groups. The predominance of energetic contributions during GIET was W_Oxi_ between both groups, including males and females in GP and A. The values of W_Oxi_ were indicated to be over 90% up to 3.5 m∙s^−1^ steps during GIET in GP and A. However, relative W_Oxi_ (%) in A was progressively decreased with increasing exercise intensity from 4.0 to 4.5 m∙s^−1^ because of the increased anaerobic contributions, such as the increased lactate production rate (a reduction in W_Oxi_ of only 8.6% from the first to the last stage). These phenomena were also confirmed in a previous study by Bertuzzi et al. [41], in which a similar incremental exercise test on the treadmill was performed with male recreational long-distance runners. Regarding gender differences in energetic contributions, males in GP exhibited absolute and relative lower W_Gly_ and higher W_PCr_ than females in GP; there were no significant differences in W_Oxi_. However, a higher trend toward W_Oxi_ was observed in male GP than in female GP, while males and females in A showed no significant differences, although there were similar tendencies to GP among the three energy systems (Figure 3A–T). Skeletal muscle mass and all fiber types are greater in males than in females. The difference between males and females is especially pronounced in type II fibers, which results in a greater ratio of type II fiber mass to type I fiber mass in males, including higher PCr pool, La^−^ production and elimination, and oxidative capacity [14,42,43,44,45]. On the other hand, greater skeletal muscle microvascular reactivity in the form of microvascular dilation and arterial diameter resulting in greater blood flow may affect gender differences in reperfusion capacity, which are also positively associated with cardiovascular fitness [14,37,46,47,48]. The abovementioned factors might differentially influence metabolic energy contributions between males and females in GP and A.

Previous studies have suggested that high aerobic conditioning comprising an aerobic base such as energy recovery is associated with improved sports performance, cardiometabolic fitness, efficient fat and carbohydrate utilization, and metabolic flexibility in adults and elite athletes. These improvements comprise decreased La^−^, CHOox, and increased FATox [5,10,13,16,17,21,49]. Performance-related jogging/running speeds were higher at all steps, and more steps were performed in A than in GP, who utilized higher FATox and lower carbohydrates during GIET.

**Figure 5:**
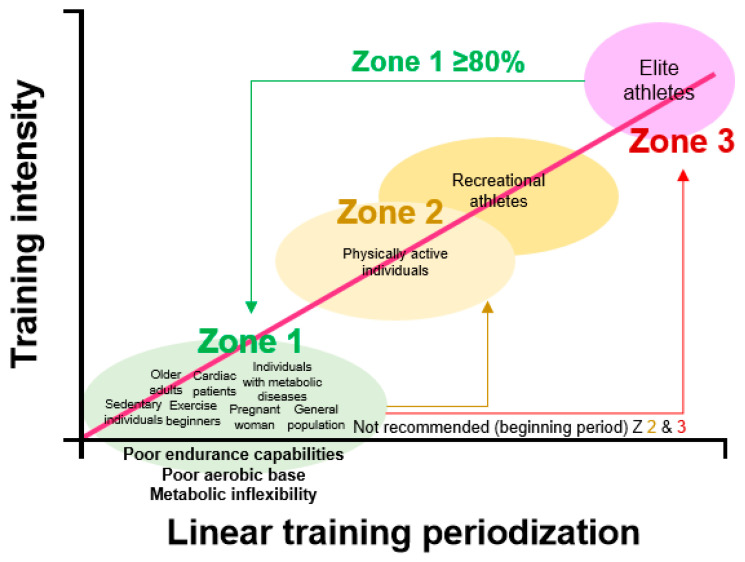
Schematic representation of training intensity zones and linear training periodization in different individuals.

In previous studies, the volume of individualized low-intensity exercises (ILIE), such as high-volume training (HVT) (zone 1, <2 mmol∙L^−1^ La^−^), were mostly performed (approximately ≥ 80%) by elite athletes such as runners and marathoners, to improve their performances during preparatory, pre-competitive, and competitive periods [49,50,51]. Furthermore, the typical linear training periodization focuses on increased aerobic base such as increased mitochondrial number, ATP re-synthesis from lactate, and capillary density [10,16,21,49]. ILIE/HVT are performed before the greater volume of high-intensity exercise is increased [21,49,52]. Most regular exercise prescriptions for the public health recommend that moderate to high-intensity physical activity/exercises be performed for at least 150 min (3–5.9 metabolic equivalents, METs) and 75 min (≥6 METs) per week [28,53]. However, sedentary individuals, cardiac patients, exercise beginners, and individuals with metabolic syndrome cannot immediately begin performing at these exercise intensities because their levels of physiological performance are low, consisting of poor FATox and directly higher utilization of carbohydrates, thus resulting in metabolic inflexibility. This was confirmed in a previous study [5] and in our present study. Recent studies have indicated that 1-h ILIE at zone 1, including a recovery domain between 4 and 9 weeks, improves the exponential lactate curve (rightward shift) in adults and professional soccer players during GIET [16,17]. In particular, Hwang et al. [17] suggest an exercise intensity at 72% from the lactate threshold 1 (before LT1), which improves the lactate curve at zones 1, 2 (moderate/threshold), and 3 (high), including the recovery domain and enhanced FATox. Therefore, the general population may perform more aerobic training at zone 1 to improve their metabolic flexibility (FATox and re-metabolism from La^−^), which can enable greater volumes at zones 2 and 3 and protect against cardiac/metabolic diseases [5,49] (Figure 5).

The current study has some limitations. Higher exercise intensity (>4 mmol∙L^−1^) is necessary, to compare CHOox in different individuals. Moreover, direct parameters regarding mitochondrial function were not measured. Therefore, further studies are needed to investigate carbohydrate availability during higher exercise intensity. Indeed, studies should examine how the same interventional approach of the abovementioned training prescription in elite athletes affects metabolic flexibility, by conducting an analysis of proteomics and metabolomics and the energetic contributions in GP and in individuals with cardiac/metabolic diseases.

## 5. Conclusions

Our findings show higher percentages of HR_max_, relative $\dot{V}$O_2mean_, $\dot{V}$CO_2mean_, METs in $\dot{V}$O_2mean_, La^−^, CHOox and lower FATox, jogging/running speeds, and GIET steps in GP than in A between certain GIET steps. Regarding energetic contributions, W_Oxi_ was predominantly utilized during GIET and W_Gly_ and W_PCr_ were greater in GP than in A, including lower W_Gly_ and higher W_PCr_ in male GP than female GP. GP showed poorer metabolic flexibility and energetic contributions as well as inefficient anaerobic contributions (glycolytic and phosphagen systems), which are associated with mitochondrial function, cardiovascular fitness, and metabolic syndrome. To achieve physical health benefits in terms of increased FATox and La^−^ elimination, along with improved oxidative capacity such as endurance performance, it is recommended that aerobic base training (baseline) as ILIE within a recovery domain (zone 1) be performed, as this is predominantly performed by elite athletes worldwide during training periods, supports higher exercise intensities (permissive of a higher volume), and may protect against risks of cardiac/metabolic diseases.

## Figures and Tables

**Table 1:** Anthropometric data at GP (*n* = 15; male: 7, female: 8) and A (*n* = 15; male: 7, female: 8) groups.

Parameters	GP	A	GP (Male)	GP (Female)	A (Male)	A (Female)
	(*n* = 15)	(*n* = 15)	(*n* = 7)	(*n* = 8)	(*n* = 7)	(*n* = 8)
Age [years]	33.13 ± 8.99	29.47 ± 7.22	36.28 ± 10.04	30.37 ± 7.52	31.85 ± 4.63	27.37 ± 8.68
Height [cm]	171.27 ± 8.50	171.67 ± 5.71	175.71 ± 7.25	167.37 ± 7.90	174.42 ± 5.02	169.25 ± 5.41
Body mass [kg]	65.49 ± 10.48	65.26 ± 6.73	71.01 ± 11.05	60.65 ± 7.61	69.64 ± 5.64	61.42 ± 5.24
Body fat [%]	16.71 ± 4.69	15.07 ± 2.32	13.84 ± 4.91	18.62 ± 3.76	14.88 ± 0.66	15.15 ± 2.90
BMI [kg∙m^−2^]	22.21 ± 2.11	22.10 ± 1.41	22.89 ± 2.41	21.60 ± 1.74	22.85 ± 0.94	21.43 ± 1.47

Data are presented as means and SD. Anthropometric data were not significantly different between GP and A. BMI: Body mass index; GP: general population; A: athletes.

**Table 2:** Physiological parameters, metabolic flexibility (fat and carbohydrate oxidation), energetic contributions during GIET (*n* = 30; GP = 15 and A = 15).

GIET	1.5 m∙s^−1^			2.0 m∙s^−1^			2.5 m∙s^−1^			3.0 m∙s^−1^			3.5 m∙s^−1^		
	GP	A	*p* (ES)	GP	A	*p* (ES)	GP	A	*p* (ES)	GP	A	*p* (ES)	GP	A	*p* (ES)
HR [beats∙min^−1^]	116 ± 13	110 ± 13	ns	133 ± 15	126 ± 15	ns	150 ± 18	139 ± 15	ns	164 ± 17	156 ± 16	**0.0319** **(*r* = −0.4)**	173 ± 18	170 ± 14	ns
% of HR_max_	63 ± 7	59 ± 7	ns	72 ± 7	68 ± 8	ns	81 ± 9	74 ± 7	**0.0299** **(*d* = 0.9)**	89 ± 8	83 ± 7	**0.0167** **(*r* = −0.4)**	94 ± 8	91 ± 6	ns
$\dot{V}$O_2mean_ [L∙min^−1^]	6.98 ± 1.18	6.39 ± 0.69	ns	8.70 ± 1.52	7.95 ± 0.87	ns	10.12 ± 1.80	9.38 ± 1.07	ns	11.65 ± 2.07	10.93 ± 1.31	ns	12.93 ± 2.09	12.51 ± 1.52	ns
$\dot{V}$O_2mean_ [mL∙kg^−1^∙min^−1^]	10.68 ± 0.84	9.82 ± 0.81	**0.0079** **(*d* = 1.0)**	13.28 ± 0.90	12.20 ± 0.78	**0.0007** **(*r* = −0.6)**	15.43 ± 0.94	14.39 ± 0.87	**0.0036** **(*d* = 1.2)**	17.77± 0.96	16.75 ± 0.95	**0.0068** **(*d* = 1.1)**	19.91± 0.95	19.17 ± 1.14	ns
METs ($\dot{V}$O_2mean_)	3.05 ± 0.24	2.81 ± 0.23	**0.0063** **(*r* = −0.5)**	3.80± 0.26	3.49 ± 0.22	**0.0009** **(*r* = −0.6)**	4.41 ± 0.27	4.11 ± 0.25	**0.0037** **(*d* = 1.2)**	5.08± 0.28	4.78 ± 0.27	**0.0068** **(*d* = 1.1)**	5.69± 0.27	5.48 ± 0.33	ns
$\dot{V}$CO_2mean_ [L∙min^−1^]	5.97 ± 1.19	5.35 ± 0.91	ns	8.02 ± 1.53	7.11 ± 1.05	ns	9.55 ± 1.82	8.46 ± 1.14	ns	11.43 ± 2.21	10.07 ± 1.39	ns	13.09 ± 2.70	11.93 ± 1.70	ns
$\dot{V}$CO_2mean_ [mL∙kg^−1^∙min^−1^]	9.12 ± 1.18	8.22 ± 1.47	**0.0367** **(*r* = −0.4)**	12.23 ± 1.12	10.92 ± 1.56	**0.0016** **(*r* = −0.6)**	14.54 ± 1.11	12.99 ± 1.55	**0.0008** **(*r* = −0.6)**	17.41 ± 1.49	15.45 ± 1.67	**0.0020** **(*d* = 1.2)**	20.07 ± 1.94	18.29 ± 2.12	**0.0378** **(*d* = 0.9)**
Glucose [mmol∙L^−1^]	4.83 ± 0.40	4.57 ± 0.31	ns	4.71 ± 0.40	4.47 ± 0.26	ns	4.40 ± 0.86	4.51 ± 0.28	ns	5.11 ± 0.63	4.56 ± 0.33	ns	5.16 ± 0.24	4.86 ± 0.63	ns
La^−^ [mmol∙L^−1^]	1.21 ± 0.31	0.81 ± 0.30	**0.0016** **(*r* = −0.6)**	1.34 ± 0.41	0.73 ± 0.22	**<0.0001** **(*d* = 1.8)**	1.86 ± 0.76	0.88 ± 0.28	**<0.0001** **(*d* = 1.7)**	3.33 ± 1.82	1.39 ± 0.58	**<0.0001** **(*r* = −0.7)**	4.80 ± 1.85	2.60 ± 1.40	**0.0017** **(*d* = 1.3)**
FATox [g∙min^−1^]	1.60 ± 0.86	1.64 ± 0.89	ns	1.26 ± 0.85	1.43 ± 0.94	ns	0.99 ± 0.92	1.53 ± 0.98	ns	0.47 ± 1.27	1.44 ± 1.19	0.0141 (*r* = −0.6)	−0.05 ± 1.33	0.99 ± 1.53	0.0159 (*r* = −0.6)
CHOox [g∙min^−1^]	4.73 ± 2.43	3.79 ± 2.58	ns	8.57 ± 2.93	6.81 ± 2.84	ns	10.96 ± 3.33	8.38 ± 2.83	**0.0304** **(*d* = 0.8)**	14.61 ± 4.69	10.74 ± 3.43	**0.0155** **(*d* = 0.9)**	18.85 ± 6.28	14.10 ± 4.44	**0.0237** **(*d* = 0.9)**
W_PCr_ [kJ]	6.37 ± 2.90	4.17 ± 1.53	**0.0186** **(*r* = −0.1)**	5.94 ± 2.44	5.95 ± 2.06	ns	6.64 ± 4.15	7.44 ± 3.20	ns	9.67 ± 7.86	7.47 ± 2.24	ns	13.74 ± 11.66	9.43 ± 5.69	ns
W_Gly_ [kJ]	1.30 ± 0.97	0.37 ± 0.75	**0.0002** **(*r* = −0.6)**	0.67 ± 0.78	0.11 ± 0.21	**0.0107** **(*d* = 0.9)**	2.17 ± 1.67	0.73 ± 0.71	**0.0046** **(*d* = 1.1)**	5.94 ± 4.39	2.05 ± 1.30	**0.0006** **(*r* = −0.6)**	9.19 ± 4.96	4.94 ± 3.48	**0.0165** **(*d* = 0.9)**
W_Oxi_ [kJ]	103.06 ± 21.55	93.59 ± 16.21	ns	139.06 ± 27.83	126.16 ± 18.39	ns	168.71 ± 32.66	156.11 ± 21.76	ns	200.74 ± 38.57	188.45 ± 25.53	ns	228.19 ± 39.33	221.57 ± 28.69	ns
W_TOTAL_ [kJ]	109.91 ± 21.66	98.91 ± 15.89	ns	144.80 ± 27.10	133.23 ± 19.16	ns	177.73 ± 32.33	164.26 ± 22.35	ns	216.34 ± 44.83	197.97 ± 26.15	ns	251.13 ± 43.07	235.93 ± 32.82	ns
W_PCr_ [%]	5.22 ± 2.75	5.18 ± 2.77	ns	3.66 ± 1.69	5.23 ± 1.80	**0.0030** **(*r* = −0.5)**	4.01 ± 2.86	4.58 ± 1.92	ns	4.30 ± 2.36	3.79 ± 1.09	ns	5.37 ± 4.22	3.88 ± 2.00	ns
W_Gly_ [%]	1.17 ± 0.78	0.35 ± 0.64	**0.0003** **(*r* = −0.6)**	0.49 ± 0.59	0.08 ± 0.16	**0.0157** **(*r* = −0.5)**	1.24 ± 0.98	0.43 ± 0.37	**0.0068** **(*r* = −0.5)**	2.76 ± 2.06	1.04 ± 0.70	**0.0048** **(*r* = −0.5)**	3.61 ± 1.96	2.05 ± 1.36	**0.0229** **(*d* = 0.9)**
W_Oxi_ [%]	93.61 ± 2.97	94.47 ± 2.66	ns	95.85 ± 1.60	94.69 ± 1.81	**0.0270** **(*r* = −0.4)**	94.76 ± 2.70	94.99 ± 2.10	ns	93.04 ± 2.45	95.17 ± 1.42	**0.0069** **(*d* = 1.0)**	91.02 ± 5.56	94.07 ± 2.82	ns

GIET: graded incremental exercise test, GP: general population, A: athletes, ES: effect size (*d* and *r*), $\dot{V}$O_2mean_: mean oxygen uptake, $\dot{V}$CO_2mean_: mean carbon dioxide, HR: heart rate, La^−^: blood lactate concentration, FATox: fat oxidation, CHOox: carbohydrate oxidation, METs ($\dot{V}$O_2mean_): metabolic equivalents in $\dot{V}$O_2mean_, W_PCr_: phosphagen system contribution, W_Gly_: glycolytic system contribution, W_Oxi_: oxidative system contribution, %: percentages. CPT: carnitine palmitoyltransferase; CHOox: carbohydrate oxidation; CoA: Coenzyme A; G 6-P: glucose 6-phosphate; PDH: pyruvate dehydrogenase; TCA: tricarboxylic acid. Data are mean ± standard deviation (SD).

## Data Availability

Data available on request due to restrictions e.g., privacy or ethical. The data presented in this study are available on request from the corresponding author. The data are not publicly available due to the privacy policy of CHA University.
